# Dexamethasone-induced cisplatin and gemcitabine resistance in lung carcinoma samples treated *ex vivo*

**DOI:** 10.1038/sj.bjc.6602453

**Published:** 2005-03-08

**Authors:** N Gassler, C Zhang, T Wenger, P A Schnabel, H Dienemann, K-M Debatin, J Mattern, I Herr

**Affiliations:** 1 Department of Pathology, University of Heidelberg, Germany; 2 Clinical Cooperation Unit Molecular Oncology, German Cancer Research Center, Im Neuenheimer Feld 280, 69120 Heidelberg, Germany; 3 Thoraxklinik-Heidelberg, University of Heidelberg, Germany; 4 Children's Hospital, University of Ulm, Germany; 5 Clinical Cooperation Unit Nuclear Medicine, German Cancer Research Center, Heidelberg, Germany

**Keywords:** lung cancer, drug resistance, cisplatin, gemcitabine, dexamethasone

## Abstract

Chemotherapy for lung cancer not only has severe side effects but frequently also exhibits limited, if any clinical effectiveness. Dexamethasone (DEX) and similar glucocorticoids (GCs) such as prednisone are often used in the clinical setting, for example, as cotreatment to prevent nausea and other symptoms. Clinical trials evaluating the impact of GCs on tumour control and patient survival of lung carcinoma have never been performed. Therefore, we isolated cancer cells from resected lung tumour specimens and treated them with cisplatin in the presence or absence of DEX. Cell number of viable and dead cells was evaluated by trypan blue exclusion and viability was measured by the MTT-assay. We found that DEX induced resistance toward cisplatin in all of 10 examined tumour samples. Similar results were found using gemcitabine as cytotoxic drug. Survival of drug-treated lung carcinoma cells in the presence of DEX was longlasting as examined 2 and 3 weeks after cisplatin treatment of a lung carcinoma cell line. These data corroborate recent *in vitro* and *in vivo* xenograft findings and rise additional concerns about the widespread combined use of DEX with antineoplastic drugs in the clinical management of patients with lung cancer.

The incidence of lung cancer has increased considerably over the past 50 years and lung carcinoma has become the leading cause of death by cancer in both men and women (Levi *et al*, 2004). The prognosis is still poor and about 80% of patients die within 1 year of diagnosis (Office for National Statistics, 1997). Despite some advances in surgical procedures, chemotherapy and radiotherapy made over the past two decades, long-term survival in, for example, non-small-cell lung cancer (NSCLC) patients at stage 3 and 4 is obtained in only 5–10% of cases (Cancer Research UK, 2004). The development of drug resistance in patients receiving chemotherapy or a combination therapy including ionizing radiation represents a major problem in cancer treatment. The present paper aims at addressing one aspect of this complex problem.

Dexamethasone (DEX) and similar glucocorticoids (GCs) were first introduced to tumour therapy on the basis of proapoptotic effects in lymphoid cells and on their effectiveness in treating tumour-related oedema, inflammation, pain and electrolyte imbalance as well as stimulating appetite, and most importantly, preventing nausea and emesis caused by cytotoxic drugs (Aapro, 1991; Cheng *et al*, 1997; Kirkbride *et al*, 2000; The Italian Group of Anticancer Research, 1995, 2000). However, prospective randomised trials evaluating a potential impact on tumour control and patient survival have never been performed.

Induction of apoptosis resistance toward chemotherapy by DEX was recently described in a cervical and a lung carcinoma cell line (Herr *et al*, 2003) as well as in a breast cancer cell line (Wu *et al*, 2004). *In vivo*, a xenografted and cisplatin-treated lung tumour cell line grew faster in the presence of DEX (Herr *et al*, 2003). These results and earlier observations of DEX-induced resistance to a broad range of cytotoxic anticancer agents (Rutz, 2002; Rutz and Herr, 2004) suggest that DEX acts proapoptotic in lymphoid but antiapoptotic in cervical, lung and breast cancer cell lines.

To analyse whether DEX might also induce cisplatin resistance in fresh tumour samples resected from patients, we treated isolated tumour cells with cisplatin or gemcitabine in the presence or absence of DEX *ex vivo*. We found that DEX promoted proliferation in all of 10 examined lung carcinomas despite the presence of cisplatin. These *ex vivo* data together with the recently published *in vitro* and *in vivo* xenograft data (Herr *et al*, 2003) suggest a need for carefully considering the use of DEX and other GCs together with cytotoxic therapy in the curative treatment of patients with lung carcinoma.

## MATERIALS AND METHODS

### Isolation of fresh tumour cells from resected lung cancer specimens

Solid tumours from fresh lung carcinomas were minced in RPMI medium supplemented with 20% heat-inactivated foetal bovine serum (Sigma, Deisenhofen, Germany), 25 mM HEPES, 2 mM L-glutamine and Pen/Strep (all from Gibco/Life Technologies, Paisley, Scotland) under sterile conditions, counted by trypan blue exclusion and immediately analysed by the MTT-assay. Patient material was obtained under the approval of the ethic committee of the University of Heidelberg. Diagnoses were established by conventional clinical and histological criteria according to the World Health Organisation (WHO). All surgical resections were indicated by principles and practice of oncological therapy. Neither GCs nor neoadjuvant chemotherapy were applicated prior to surgery. Tumour types and stages are shown in the Table 1.

### Cell culture

The established P693 lung carcinoma cell line (NSCLC) is described and was grown at 37°C in DMEM (Life Technologies Gibco BRL, Karlsruhe, Germany), supplemented with 10% foetal bovine serum (Life Technologies).

### Drugs

A stock solution of cisplatin (Sigma) was prepared in DMSO at the concentration of 33 mM. Gemcitabine (kind gift from Eli Lilly, Indianapolis, IN, USA) was diluted in PBS to a 50 *μ*M stock. A 25 mM stock of DEX (Sigma) was prepared in ethanol. Final concentrations of the solvents in medium were 0.01% or less. We used DEX in concentrations between 0.1 and 10 *μ*M since these amounts are comparable with the clinical setting where one dose of 12 mg DEX (about 0.15 mg kg^−1^) resulted in plasma concentrations of 0.25–0.5 *μ*M with a half-life of several hours (Brady *et al*, 1987). We employed cisplatin in concentrations of 7–34 *μ*M, which are within the range found in tumours and plasma of patients treated with a single dose of 150 mg m^−2^ cisplatin (Tegeder *et al*, 2003).

### MTT assay

Freshly isolated tumour cells were resuspended at a final concentration of 5 × 10^5^ ml^−1^ and plated in 96-well microplates, 100 *μ*l per well. Dexamethasone was added immediately and the microplates were maintained in a 5% CO_2_ incubator. Cisplatin or gemcitabine were added 24 h later. At 48 h after incubation of cisplatin 10 *μ*l of a 10 mg ml^−1^ MTT-solution (3-[4,5-dimethyl thiazo-2-yl]-2,5-diphenyltetrazolium bromide, from Sigma) were added. At 8 h later medium was replaced by dimethyl sulphoxide (Serva, Heidelberg, Germany) followed by incubation at 37°C for 5 min. The optical density of wells (intensity of blue colour depending on mitochondrial activity) was measured by an ELISA reader at 550 nm wavelength.

## RESULTS AND DISCUSSION

**Figure 1:**
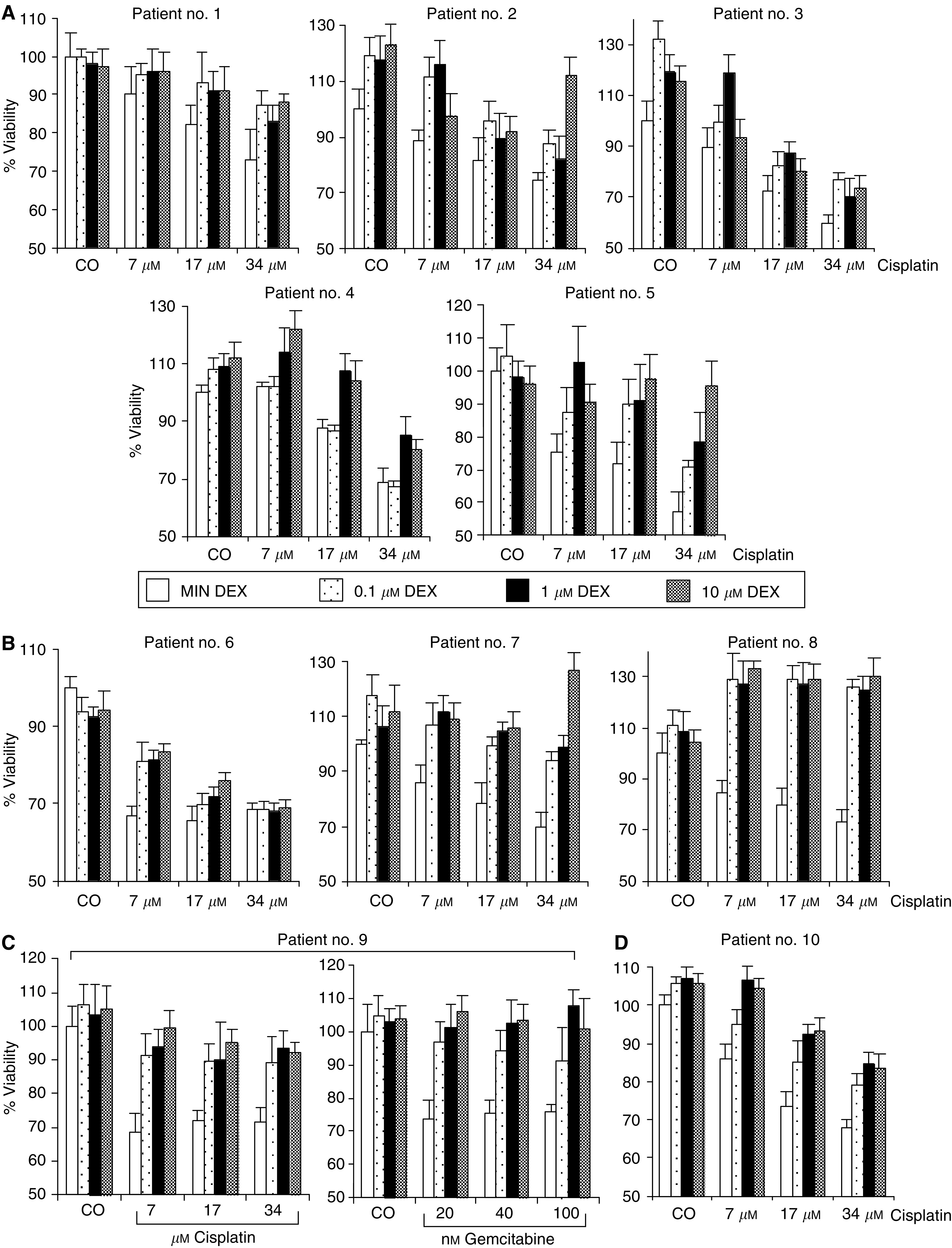
DEX promotes cisplatin- and gemcitabine-inhibited viability of fresh lung carcinoma sample. Tumour cells from (**A**) five adenocarcinomas (**B**, **C**) four squamous cell carcinomas and (**D**) one small cell carcinoma were isolated and were cultivated in a concentration of 5 × 10^5^ ml^−1^ in the absence (white bars) or presence of DEX (0.1, 1 or 10 *μ*M as indicated) for 24 h. Thereafter, cisplatin or gemcitabine were added in concentrations indicated or not (CO) and viability was measured by the MTT-assay after additional 48 h. Eight wells per treatment were analysed and s.d. were less than 10%.

**Figure 2:**
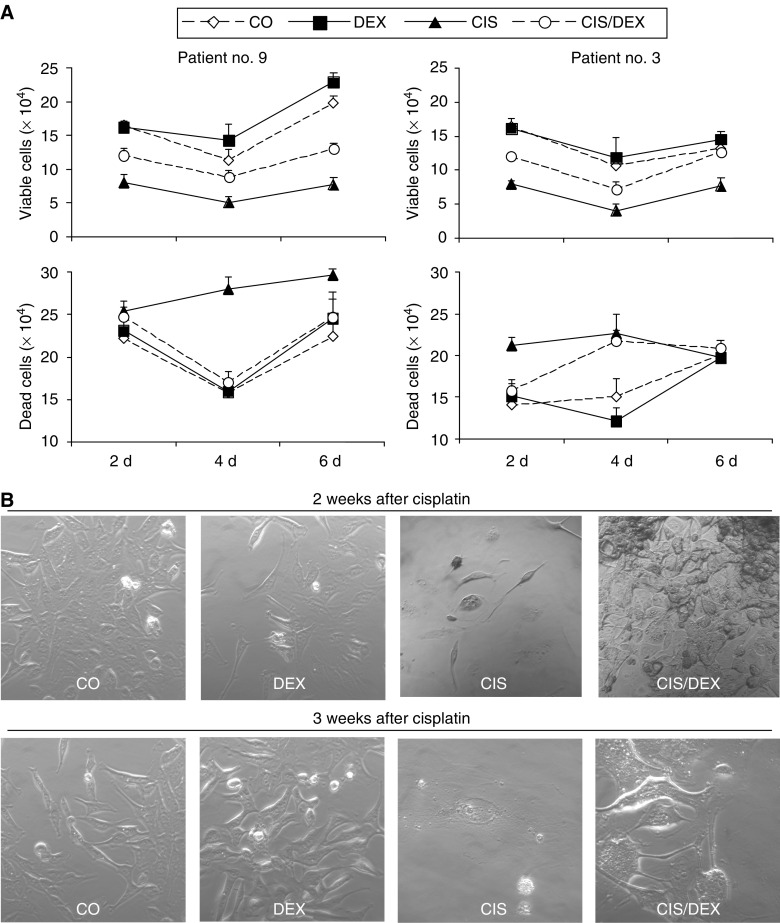
DEX promotes proliferation and inhibits drug-induced death of fresh lung carcinoma samples. (**A**) Freshly isolated tumour cells from patient 3 and 9 were cultivated in a concentration of 5 × 10^5^ ml^−1^ in the absence or presence of DEX (1 *μ*M) for 48 h as indicated. Cisplatin (CIS, 17 *μ*M) was added or not as indicated and viable or dead cells were counted by trypan blue exclusion 2, 4 or 6 days (d) later. s.d. are shown and were less than 10%. (**B**) P693 lung carcinoma cells were treated as described above and cells were photographed 2 and 3 weeks later.

Since recent findings describing DEX-induced therapy resistance in human carcinomas including lung cancer (Herr *et al*, 2003; Rutz, 2002; Rutz and Herr, 2004) may be relevant for the treatment of patients, we studied the effect of DEX on tumour cells freshly isolated from resected lung tumour specimens. Cisplatin strongly reduced cell viability in all carcinoma samples derived from patients 1 to 10, whereas the presence of DEX prevented this effect (Figure 1a–d). These data include five adenocarcinomas (Figure 1a), four squamous cell carcinomas (Figure 1b, c) and one small cell carcinoma (Figure 1d, compare also Table 1). Furthermore, DEX prevented also the cytotoxic effect of gemcitabine (Figure 1c). To examine whether this effect might be due to enhanced proliferation or inhibited cell death, we counted viable and dead tumour cells 2, 4 and 6 days after treatment with cisplatin in the presence or absence of DEX. Whereas cisplatin reduced the amount of viable cells and enhanced the amount of dead cells, the presence of DEX inhibited this effect as shown for patient no. 9 and 3 (Figure 2a). Since primary lung tumour cells are not suited for long-term studies, we examined long-term survival using the NSCLC cell line P693. Cells were treated with cisplatin in the presence or absence of DEX. After 2 and 3 weeks, cells were photographed and counted by trypan blue exclusion. After 2 weeks more than 90% and after 3 weeks 100% of the cells treated with cisplatin alone were dead. Cells treated with cisplatin in the presence of DEX were swollen and showed typical features of necrotic cell death. However, these cells recovered and started to grow again 3 weeks after cisplatin (Figure 2b).

GCs are known to exert proapoptotic and antiproliferative effects in lymphoid cells, and to reduce many of the symptoms caused by tumours as well as their treatment. Hence, these agents are widely used in cancer therapy including the highly effective synthetic GC DEX (Rutz, 2002; Rutz and Herr, 2004). Our present data demonstrate induction of resistance toward cytotoxic therapy in lung cancer samples by *ex vivo* cotreatment with GCs. We report here that DEX blocked the therapeutic effect in tumour cells isolated from resected lung cancer specimens. These results are not specific for cisplatin since DEX inhibited also gemcitabine-induced apoptosis in our studies, which confirm recent data found in a lung cancer cell line (Bergman *et al*, 2001). Also, DEX has been shown to induce resistance toward diverse other cytotoxic agents used in cancer therapy and also in many different malignant cell types *in vitro* (Rutz, 2002; Rutz and Herr, 2004).

Furthermore, DEX enhanced basal viability in most of the 10 examined lung tumour samples, whereas basal viability was inhibited in one sample and no effect was seen in another one. We actually do not know the reason for these variations *ex vivo*, but assume that they might be due to intrinsic properties of the tumours and the quality of preparations of lung carcinoma cells. Therefore, clearly more *ex vivo* experiments and clinical studies are really needed. However, the results are so potentially very important for the outcome of therapy that we feel they have to be reported now.

The mechanisms by which GCs induce apoptosis in lymphoid cells are well studied. These include depolarisation of the mitochondrial membrane potential, enhanced expression of the death receptor CD95 and its ligand, followed by activation of the caspase cascade (Kofler, 2000; Planey and Litwack, 2000; Distelhorst, 2002). The same mechanisms that are induced in lymphoid cells have been shown to be blocked in several carcinoma cells by GCs, thereby inhibiting chemo- and radiatio-induced apoptosis (Herr *et al*, 2003; Wu *et al*, 2004). An open question is, how do GCs mediate these cell-type specific effects that have been clearly shown to be related to a functional glucocorticoid receptor (GR) (Herr *et al*, 2003; Wu *et al*, 2004). An explanation for the cell-type specific antiapoptotic properties of GCs may be the differential expression of GR coactivators and corepressors in diverse cell types, as proposed to explain the opposite effects of tamoxifen on mammary *vs* endometrial tissue (Shang and Brown, 2002). A recent study compared gene expression of a breast cancer cell line with genes found to be regulated by DEX in lymphocytes (Wu *et al*, 2004). Surprisingly, only a few of the genes regulated by DEX in carcinomas are the same as those identified as GC-regulated in lymphocytes. Among the differentially regulated set of sequences are apoptotic genes as well as genes involved in signal transduction, metabolism, transcription, cell cycle, DNA repair and others (Rutz, 2002; Rutz and Herr, 2004). These data strongly suggest that tissue-specific differences in GC-induced apoptosis *vs* survival outcomes may be due to cell-type-specific transcriptional regulation.

In conclusion, we have shown that application of DEX renders lung cancer cells from fresh tumour tissue and not only established carcinoma cell lines that have undergone a selection process resistant to cytotoxic therapy with cisplatin and gemcitabine. This finding urges carefully reconsideration of the widespread use of GCs in treatment protocols for patients with lung cancer. Alternatively, nonsteroidal agents such as serotonin-receptor antagonists or the new NK1 receptor antagonists may be sufficient to effectively manage at least some of the tumour and treatment related symptoms, particularly, nausea and emesis.

## Figures and Tables

**Table 1:** Characterisation of lung tumour tissue from patients 1–10

**Patient no.**	**Gender**	**Age (years)**	**Histological typing (WHO)**	**pTNM**
1	M	70	Adenocarcinoma	pT2N2M0 G3
2	M	60	Adenocarcinoma	pT2N0M0 G2
3	M	54	Adenocarcinoma	pT3N0M0 G3
4	F	64	Adenocarcinoma	pT2N2M0 G3
5	M	47	Adenocarcinoma	pT4N2M0 G3
6	M	60	Squamous cell carcinoma	pT3N3M0 G3
7	M	75	Squamous cell carcinoma	pT4N0M0 G2
8	F	45	Squamous cell carcinoma	pT2N0M0 G3
9	M	55	Squamous cell carcinoma	pT2N0M0 G3
10	F	66	Small cell carcinoma	pT2N2M0 G3

M=male; F=female.
